# 

**Published:** 1850-09

**Authors:** 


### NOTICE TO CORRESPONDENTS.

Communications and Books for notice should be addressed to the Editor, care of Messrs. Lindsay & Blakiston.

Letters, &c., connected with the *business affairs* of the Journal should be addressed to the Publishers.

Papers for publication must be received *before* the 20th of the month, or they cannot appear in the forthcoming number.

The following Journals have been received in exchange:

The Boston Medical and Surgical Journal. (Weekly, Boston.)
Buffalo Journal.
Medical News.
Western Lancet.
Western Jourual.
North-Western Medical and Surgical Journal.
The British American Journal of Medical and Physical Science. (Montreal.)
The London Lancet. (Weekly, London.)
The Medical Times. Weekly, London.
Dublin Medical Press.
Provincial Medical and Surgical Journal.
British and Foreign Medico-Chirurgical Review. July.
Edinburgh Monthly Journal. July.
Edinburgh Medical and Surgical Journal.
Dublin Quarterly Journal. April and July.
London Medical Gazette. April.
Pharmaceutical Journal.
Braithwaite's Retrospect.
London Journal of Medicine.
Ranking's Abstract.

—

The following works have also been received for notice:
Mitchell's Therapeutics. From Lippincott & Grambo.
Dunglison's Physiology, 4th edition. From Lea & Blanchard.
Copland on Apoplexy and Palsy. From the same.
Howard on the Eye.
Spectacles, their Uses and Abuses. By Sichel.

—

Communications have been received from:—
W. J. Reese, M. D., Alabama.
William Waters, M. D., Fredericktown, Md.
G. H. H. Koontz, Woodstock, Va.
E. C. Banks, Lawrenceville, Ill.
H. A. Ramsay, M. D., Raysville, Ga.
C. Hixson, Student of Mediciue, Middletown, Conn.

—

The foreign correspondents of the Examiner will please direct their Exchanges and other communications to the care of Mr. Charles J. Skeet, 27 King William St., Charing Cross, London, or Mr. H. Bossange, 21 Bis, Quai Voltaire, Paris.

